# Pregnancy Rate after Controlled Ovarian Hyperstimulation and Intrauterine Insemination for the Treatment of Endometriosis following Surgery

**DOI:** 10.1155/2015/282301

**Published:** 2015-07-12

**Authors:** Attila Keresztúri, Zoltan Kozinszky, József Daru, Norbert Pásztor, János Sikovanyecz, János Zádori, Virág Márton, Sándor Koloszár, János Szöllősi, Gábor Németh

**Affiliations:** ^1^Department of Obstetrics and Gynecology, Faculty of General Medicine, Albert Szent-Györgyi Medical Center, University of Szeged, Szeged 6725, Hungary; ^2^Reproductive Medicine, Department of Obstetrics and Gynecology, Karolinska University Hospital Huddinge, Stockholm, Sweden; ^3^Center for Assisted Reproduction, Kaáli Institute, University of Szeged, Szeged 6723, Hungary

## Abstract

*Objective*. To compare pregnancy rate after controlled ovarian hyperstimulation and intrauterine insemination (COH-IUI) with no treatment in patients with endometriosis-associated infertility treated with laparoscopy. *Design*. A clinical cohort study. *Setting*. University-level tertiary care center. *Patients*. 238 women with various stages of endometriosis after laparoscopic treatment. *Interventions*. Either COH-IUI or follow-up for 12 months. *Main Outcome Measures*. The primary outcome measures were clinical pregnancy and live birth rate. Predictive factors evaluated were female age, maternal BMI, and duration of infertility. *Results*. The pregnancy rate attained after the integrated laparoscopy–COH-IUI approach was 53.4%, while it was significantly lower (38.5%) in the control group. Similarly, a significant difference was observed in live births (48.3% versus 34.2%). Patients with severe endometriosis were less likely to achieve pregnancy (38%) and live birth (35%) than their counterparts with milder forms (57% and 53%). *Conclusions*. In patients with endometriosis-based infertility, surgery followed by COH-IUI is more effective than surgery alone.

## 1. Introduction

Endometriosis, the leading cause of infertility, has a prevalence of 0.5–5% in fertile women and 25–40% in infertile women [1–3]. Although a broad spectrum of therapeutic options is available, evidence-based management of infertility associated with endometriosis is still disputed [3–5].

Studies have provided conflicting results on surgery and endometriosis. Although the majority of reports have indicated that reparative surgery improves the rate of pregnancy significantly, further research is needed on the efficacy of surgery in endometriosis [1, 6]. Marcoux et al. [7] reported that operative laparoscopy provides a significantly elevated monthly fecundity rate compared to diagnostic laparoscopy in endometriosis, while Parazzini et al. [8] found no significant improvement in the 12-month cumulative pregnancy rates in stage I-II endometriosis. Women with advanced endometriosis have a cumulative pregnancy rate of 44% after 12 months and 57.5% after 24 months, which is significantly more favorable for stage III endometriosis patients than for those with stage IV following surgery [9].

A combination of controlled ovarian hyperstimulation (COH) with intrauterine insemination (IUI) has been evaluated for the treatment of infertility in surgically corrected endometriosis [3, 5, 10], though the success of COH-IUI is lower in endometriosis than in other types of infertility [5, 11]. COH-IUI after the surgical removal of endometrial deposits improves the success rate less than in vitro fertilization (IVF) [1, 3, 6].

The effect on pregnancy odds of COH-IUI after a combination of laparoscopic resection/ablation of the endometriotic lesions is little documented with conflicting results [12–15]. The aim of our present study was therefore to assess whether COH-IUI has a significant effect on pregnancy rate (PR), cumulative pregnancy rate (CPR), and live birth rate (LBR) in infertile patients with endometriosis after laparoscopic surgery.

## 2. Materials and Methods

This prospective clinical cohort study was conducted at the Department of Obstetrics and Gynecology, University of Szeged, from January 2002 to December 2008. All infertile couples who presented at our infertility ward participated in a complete investigation related to patient history, hysterosalpingogram, serum endocrine profile in the follicular phase, midluteal progesterone concentrations, and sperm analysis of the male partner.

### 2.1. Inclusion and Exclusion Criteria

Eligibility criteria were as follows: the couple not conceiving after at least 1 year of unprotected intercourse; the women being younger than 40; confirmation of an ovulatory cycle by midluteal serum progesterone level; symptoms suggestive of endometriosis (severe dysmenorrhea; deep dyspareunia; chronic pelvic pain; ovulation pain; perimenstrual bowel- or bladder-associated symptoms with or without abnormal bleeding; and chronic fatigue); clinical signs (pelvic tenderness, a fixed (retroverted) uterus, tender uterosacral ligaments, or enlarged ovaries); incremental sonographic finding (endometrioma(s)); and a normal spermiogram of the partner according to the World Health Organization criteria (sperm concentration at least 20 million/mL and 50% progressive motile spermatozoa within 1 h of ejaculation) [16]. Couples who presented with other gynecological pathologies or coexisting causes of infertility besides endometriosis were excluded. Women with additional obvious causes of infertility such as any abnormality in anatomy, tubal factor, ovulatory dysfunction, and polycystic ovarian syndrome were also excluded from the study. Previous COH-IUI treatment or steroid therapy and any condition affecting ovarian function or blood-clotting disturbance that would exclude patients from COH-IUI treatment (early menopause and ovarian malignancy) also gained ground as exclusion criteria.

### 2.2. Patient Recruitment

After baseline registration, 382 patients were approached for potential participation, and 68 women declined to participate. In some patients with endometriosis, surgery may have been indicated to treat pain. Women were excluded at this stage if they were medically unfit for laparoscopy due to other diseases (*n* = 1) or previous or current gynecological malignity (*n* = 1). Another exclusion criterion was an inability to communicate in Hungarian (*n* = 1). The participants were informed of the study in detail and signed their informed consent to participation.

All eligible participants (*n* = 311) were referred for day-care operative laparoscopy for removal of endometriotic lesions and adhesiolysis when necessary. At this stage, 47 participants were excluded as endometriotic lesions were not found in the abdominal cavity during surgery. 26 women were excluded because chromopertubation with methylene blue dye revealed tubal patency as a cause of infertility besides endometriosis.

In the remaining 238 cases, various stages of endometriosis were identified by direct visualization, in accordance with the revised American Society for Reproductive Medicine classification [17]: 218 patients underwent laparoscopic surgery and 20 patients were managed by laparotomy. Salpingectomy was not performed. In 98 patients, an ovarian endometrioma was removed, and cystectomy was associated with adhesiolysis in 68 cases. In 198 patients, peritoneal implants were electrocoagulated with bipolar forceps. In all the patients, biopsy specimens were taken during surgeries, and diagnosis was confirmed by histopathologic examination.

**Figure 1:**
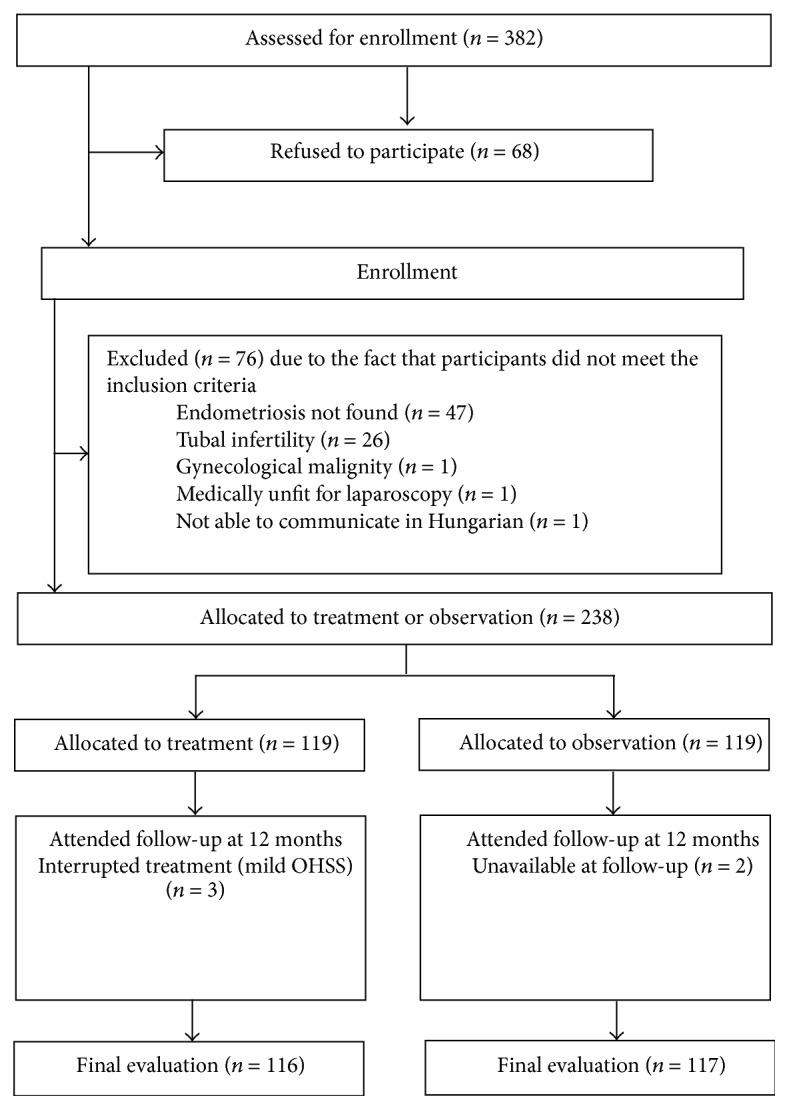
Study flow chart.

Current demographic data and clinical characteristics were recorded after operation with the exception of the duration of the menstrual cycle and infertility, which were registered at the initial presentation before surgery. This was followed by allocation of the participants into a case group (COH-IUI intervention) or an equally large follow-up group (observation without treatment). Nonrandom allocation was based on age, BMI, and stage of endometriosis in order to obtain two satisfactorily comparable matched study groups. Both study groups underwent the same surgery protocol for endometriosis. Excision of endometriotic lesions was performed in 118 cases, cystectomy and adhesiolysis were performed in 49 cases, and 98 patients underwent electrocoagulation of the endometriotic deposits in the COH-IUI treatment group, while 120 patients underwent resection, 49 received cystectomy with or without adhesyolysis, and 100 were electrocoagulated in the expectant management group. Surgery was performed by the same authors in both groups (Attila Keresztúri, József Daru, János Szöllősi, and Sándor Koloszár).  Figure 1 contains a flow chart for the study.

### 2.3. Controlled Ovarian Hyperstimulation, Semen Preparation, and IUI

In the treatment group, COH was started according to the monofollicular protocol and was initiated in the first menstrual cycle after the operation. The patients were given clomiphene citrate (CC) 100 mg/day (Clostilbegyt; Egis, Hungary) between days 3 and 7 of the menstrual cycle. From day 5 one ampoule Merional (IBSA Pharma, Slovakia, 75 IU highly purified human menopausal gonadotropin (hMG) containing follicle-stimulating hormone (FSH) with luteinizing hormone (LH)) was administered intramuscularly every other day. When there was no sonographic evidence of ovarian follicular activity and serum estradiol was <60 pg/mL, the dose of Merional was doubled every other day. On day 5 or 6 of the stimulation, a transvaginal ultrasound (TVUS) examination was carried out. If the size of the dominant follicle and/or the thickness of the endometrium did not reach the required size, the administration of Merional was continued, with daily TVUS examination. When the dominant follicle reached ≥17 mm and the endometrium was thicker than 9 mm (consisting of 3 layers), 10,000 IU human chorionic gonadotropin (hCG; Choragon, Ferring) was given for luteinization after a determination of serum estradiol level. Whenever a risk of ovarian hyperstimulation syndrome (OHSS) was suspected (e.g., >10 follicles, >2 dominant follicles, ovary size > 10 cm, or a serum E2 level > 6000 pmol/L), luteinization was not carried out and cycles with premature luteinization (progesterone levels > 1 ng/mL on the day of hCG) were also ruled out. Patients that had undergone COH but had not been inseminated were excluded from the study. Standard protocols were used for insemination, which are detailed in a previous study [18]. In brief, insemination involved extraction of the concentration of sperm from the homogenized semen. Ejaculates, obtained by masturbation, were prepared for IUI. The sperm was washed and enriched by means of swim-up technology. When the concentration reached the 40 × 10^6^/mL value, 3 mL of washing fluid (Spermfit, Biomedical) was added to 1 mL of ejaculate. 400 g of the mixture was centrifuged for 10 minutes, and the supernatant was drawn off.

Double IUI procedures were performed 36 hours after hCG administration and on the subsequent day. All IUI procedures were performed by the same four authors (Attila Keresztúri, József Daru, János Szöllősi, and Sándor Koloszár). To achieve the best possible effect in the IUI group, intercourse was forbidden 5 days before the procedure and for the rest of the cycle afterwards. The luteal phase was not supported by any medication.

### 2.4. Patient Follow-Up

A serum hCG test was performed to confirm pregnancy at the time of the first expected menstrual period. Clinical pregnancy was diagnosed 2 weeks after a positive test by ultrasound, indicating the presence of an intrauterine gestational sac with a fetal echo. COH-IUI treatment continued for a maximum of 6 cycles up to successful pregnancy, with a no-treatment interval of 1-2 months interposed, which is acceptably effective [12].

The follow-up period lasted for 12 months from the surgery in both study groups. The patients in the follow-up group were referred for COH-IUI, while the patients who had undergone insemination and did not achieve pregnancy after the follow-up period were referred for IVF and embryo transfer (IVF-ET). Our primary end point was pregnancy. Secondary end points were overall cycle fecundity and live birth. Miscarriage was defined as nonvital pregnancy or the loss of a previously visible pregnancy, while an ectopic pregnancy was a conception outside the uterine cavity.

### 2.5. Statistical Analyses

Statistical analysis was performed with the *t*-test and chi-square test as appropriate. The data were analyzed with the Statistical Package for the Social Sciences (SPSS) for Windows (version 15.0; SPSS, Inc., Chicago, IL, USA). For sample size calculation, we used StatMate 2 for Windows software. Assuming a PR of 11% [13] following COH-IUI, it was calculated that 220 cycles would be required to detect an absolute 5% increase with an alpha error level of 0.05 and a beta error level of 0.2. The crude probabilities of pregnancy and live birth rates were calculated with the starting point of surgery. Cumulative ongoing clinical pregnancy rates are illustrated for both groups. To test between-group differences, the Kruskal-Wallis analysis of variance was used. Statistical significance was set at *p* < 0.05 and all statistical tests were 2-sided. The study protocol was approved by the Clinical Research Ethics Committee at the University of Szeged.

## 3. Results

Out of the 238 women eligible for inclusion, 119 were allocated into the treatment group and 119 into the follow-up group. One participant in the control group was lost to follow-up, and 3 women did not take part in hormonal induction because of the development of mild OHSS (Figure 1). The baseline values were used as follow-up values in the statistical analysis.

There were no significant differences between the two groups concerning age, duration of infertility, body weight, BMI, distribution of endometriosis stages, and sperm parameters. Endometriosis was found in stage I or II in 100 patients (42.0%) and in stage III or IV in 138 patients (58.0%). The patients were between 23 and 39 years of age (means: 33.5 years versus 32.9 years in the case and follow-up groups, resp.), and the lengths of ongoing infertility were 3.1 years and 2.9 years in the case and follow-up groups, respectively. Both the body weight (means: 65.2 kg versus 64.0 kg, in the case and follow-up groups, resp.) and the BMI (means: 22.2 kg/m^2^ versus 21.9 kg/m^2^, in the case and follow-up groups, resp.) were similar in the two groups. The duration of the menstrual cycle ranged from 24 to 39 days in both groups (means: 27.2 days versus 29.3 days, in the case and follow-up groups, resp.). Sperm qualities have also been tabulated (Table 1).

Table 2 provides an overview of the clinical characteristics of the COH-IUI cycles. Both the hormone profiles on day 3 and the hormone profiles and endometrium thicknesses on the day of hCG administration were appropriate. The IUI protocol was cancelled in only 3 cases due to ovulation induction, with CC+hMG causing mild OHSS.

Table 3 relates to the PR and LBR in the two groups. The 116 treated cases underwent 281 cycles of COH-IUI, with a mean of 2.42 cycles per patient. 62 pregnancies occurred, thus representing an overall cycle fecundity of 22.1% and a CPR of 53.4%.

Forty-five pregnancies were recorded among the 117 control participants. The CPR of 38.5% was significantly lower than that among the COH-IUI cases (53.4%). The CPR in the first 6 months was 27.3%, with a further increase of 11.1% in the following 6 months, which is significantly different (*p* < 0.05).

**Figure 2:**
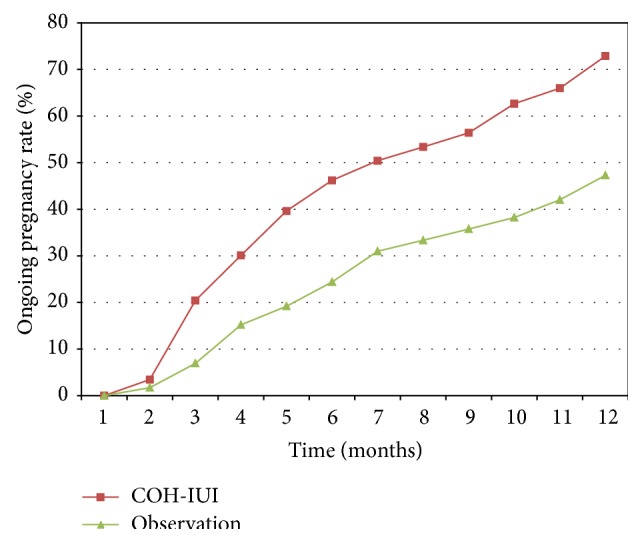
Cumulative ongoing clinical pregnancy rates in the two study groups at the Department of Obstetrics and Gynecology, University of Szeged, from January 2002 to December 2008.

The CPR in stages I-II and stages III-IV in the COH-IUI group were 64.6% and 45.6%, respectively. The corresponding rates in stages I-II and stages III-IV in the follow-up group were 50.0% and 29.9%, respectively; the differences did not reach the level of significance. The CPR was significantly higher among those in stages I-II (64.6%) than in those with endometriosis stages III-IV (45.6%) (*p* = 0.005). The Kruskal-Wallis test revealed a significant difference (*p* = 0.003) in the cumulative ongoing pregnancy rates between the two groups (Figure 2), indicating that the COH-IUI intervention significantly increased the likelihood of pregnancy relative to that in the follow-up group during the one-year study period.

Among the total of 107 pregnancies, 5 spontaneous abortions (4.7%) and 3 ectopic pregnancies (2.8%) were recorded, and there was a higher LBR in the COH-IUI group (48.3%) than in the follow-up group (34.2%). Four participants had twins after COH-IUI (6.5%). A previous diagnosis of endometriosis at stage I or II was associated with a significantly higher LBR (57.1%) than that for stages III-IV (37.8%) (*p* = 0.005).

As expected, patients with endometriosis tended to have lower odds of pregnancy as the age increased (*p* = 0.04, *r* = 0.62) and at higher BMI (*p* = 0.001, *r* = 0.73). The PR was significantly lower among women over 35 years of age than among younger women (24.6% versus 53.8%). The PR per cycle in women over 35 (12.3%) was similarly significantly lower than in younger women (23.1%). To our surprise, the duration of infertility was not a significant predisposing factor for achieving pregnancy in endometriosis (*r* = 0.21). There was no recognizable association between the sperm parameters or endometrium thickness and the success of COH-IUI.

## 4. Discussion

Our most striking finding is that the probability of conception and a resultant live birth is significantly higher after integrated laparoscopy–COH-IUI management than that of spontaneous pregnancy and live birth after surgical treatment only in women with endometriosis and particularly among those with a mild form.

Twelve-month cumulative pregnancy rates in stages I-II were 50% for spontaneous attempts and 64.6% for COH-IUI and in stages III-IV were 29.9% for spontaneous attempts and 45.6% for COH-IUI in our study. Although these differences are not significant, they suggest considerably elevated pregnancy odds for COH-IUI compared to expectant management. Cumulative pregnancy rates for COH-IUI in stages I-II were significantly higher than in stages III-IV. Moreover, live birth rates are slightly more frequent following a COH-IUI procedure than in the group with spontaneous attempts during 12 months.

Based on our results, COH-IUI should therefore be offered to patients as a treatment option after laparoscopy, particularly in moderate-to-severe forms, in agreement with other studies [19, 20] and in contrast with one study [15]. Gandhi et al. [15] stated that stimulation with gonadotropins is superior to stimulation with CC.

The latest retrospective report has demonstrated poorer results on the efficacy of postoperative COH-IUI in endometriosis, where the cumulative pregnancy rate after 6 COH-IUI (12 months) was 45% and 42% in controls in mild-to-moderate endometriosis and the corresponding rates were 10%–20% in advanced endometriosis, respectively [15]. These rates are somehow lower than our results, but there were far fewer participants included in the study and the results reported were mixed for COH only, IUI only, and COH-IUI cycles. Furthermore, they mostly used only CC/Letrozole as COH agents [15] and not CC/hMG, as we did.

Endometriosis has the lowest likelihood of fertility among infertile patients [5, 11], with the odds of fecundity in untreated women with endometriosis ranging from 0.02 to 0.1% [21]. This is enhanced after laparoscopic treatment from 30–37% at 2 years [7, 22] to 60% at 3 years [23]. Our results revealed that COH-IUI results in a PR per cycle of 22.1% after surgery. A minor limitation of our study is that a matched control study was performed rather than a randomized controlled trial (RCT). No RCT has been published on the effectiveness of COH-IUI treatment after surgery. Furthermore, COH-IUI is an advocated treatment option for minimal and mild endometriosis [3, 6], and there is a relative dearth of reports on the fecundity rate of COH-IUI after reparative surgery of moderate and advanced stages [1].

It has been concluded by others [12, 13] that a course of COH-IUI up to 3 cycles is the optimal regimen. However, our data suggest that 6 cycles appear beneficial. Furthermore, there is no consensus in the literature on prioritizing single or double IUI; the reported trend is for better results with double COH-IUI [24], and the double procedure was therefore applied in our routine.

Although the power of our study is not sufficient for a comparison of the endometriosis subgroups, it seems that women with more severe endometriosis were less likely to have a successful reproductive outcome from COH-IUI than those with mild disease. In the cumulative pregnancy curve (Figure 2), it should be noted that the immediate postoperative period was particularly favorable for conception in the follow-up group. Moreover, as expected, a significant inverse correlation was observed between disease severity and spontaneous pregnancy. In contrast, among women with stage I or II endometriosis, the fecundity rate remained elevated throughout the first year after surgery, whereas patients with endometriosis at advanced stages had a significantly higher fecundity in the first half year than in the second (*p* < 0.05). This suggests that, after combined therapy with surgery treatment, the ideal waiting time would be half a year for endometriosis in an advanced stage. This contrasts somewhat with a proposal made following a recent meta-analysis where 6–18 months was suggested for spontaneous pregnancy [1].

Our results revealed that stimulation with CC/hMG resulted in a higher CPR than that with CC [15]. As in the literature [5], the rate of twins (6.5%) and the frequency of OHSS (2.5%) were satisfactorily low in our sample, which is characteristic of endometriosis.

Several reports have described factors leading to poor prognosis of COH-IUI as an infertility diagnosis [1, 3–6, 11], in accordance with our results, including advancing female age [1, 3, 4, 6, 11], a higher BMI [11, 25], and endometriosis itself. Inconsistent with previous reports, a longer infertility period [5, 11, 25] did not decrease the likelihood of conception in our dataset, and the success of COH-IUI was not influenced by endometrial thickness above 9 mm [25], a poorer sperm motility [11, 25], or a lower content of inseminated sperm [11, 25] in our patients with endometriosis. Moreover, our study comprised selected patients with infertility, while most of the previous reports included women without infertility before the laparoscopy, which could bias the studies.

The data on surgical treatment of advanced endometriosis with the aim of pregnancy are inconclusive [1, 7, 9, 22, 23, 26]. In cases of ovarian endometrioma-associated infertility, surgery must be considered as the first-line treatment, whatever the subsequent proposed technique [22]. Some studies propose assisted reproductive technologies without prior surgery in stage IV endometriosis, due to excessive ovarian damage during surgery leading to a decreased ovarian reserve and response and thus resulting in a lower PR [27, 28]. It has also been established that the CPR is significantly higher for IVF-ET than for COH-IUI (73% versus 41%), especially in stage IV and in women over 38 years of age [13] when prior surgery has not occurred. If COH-IUI is attempted, it should not exceed three to four cycles, and a COH-IUI failure beforehand does not affect the IVF-ET outcome adversely [13].

A recent study [14] suggests that ovarian stimulation and IUI in women with surgically treated mild endometriosis is as effective as in unexplained infertility. Werbrouck et al. [14] reported a PR per cycle of 21% in minimal endometriosis and of 18.9% in the mild stage, following COH-IUI shortly after laparoscopic excision, which is comparable to our result (22.1%). However, the cumulative LBR within 4 cycles of COH-IUI was higher in women with minimal or mild endometriosis (70.2% and 68.2%) [14] as compared with our results (62.5% versus 44.5%), but only 4 double IUI cycles were used in regimen and only patients with no previous pregnancy were included in our study.

## 5. Conclusions

In conclusion, after operative treatment of any type of endometriosis, COH-IUI is indicated as a second-line treatment even in cases of advanced maternal age. The combined approach of surgery and IUI may offer improved chances of pregnancy for infertile women with endometriosis. Patients should be advised to begin attempting to conceive naturally soon after laparoscopic surgery. When pregnancy does not occur within 6 months, they should move on to COH-IUI. Further research is warranted to provide further evidence for the role of postoperative COH-IUI in fertility treatment for endometriosis.

## Figures and Tables

**Table 1:** Baseline characteristics of participants in the COH-IUI group (*n* = 116) and the control group (*n* = 117) at the Department of Obstetrics and Gynecology, University of Szeged, from January 2002 to December 2008.

	Treatment group (COH-IUI) (*n* = 116)	Control group (*n* = 117)	*p* value	OR (95% CI)
Age (in years) (mean ± S.D.)	33.5 ± 4.2	32.9 ± 4.4	NS	
Duration of menstrual cycle (days) (mean ± S.D.)	27.2 ± 3.4	29.3 ± 6.2	<0.05	
Endometriosis				
Stage I or II	48 (41.4%)	50 (43.7%)	NS	0.95 (0.56–1.60)
Stage III or IV	68 (58.6%)	67 (57.3%)		
Body weight (kg) (mean ± S.D.)	65.2 ± 7.3	64.0 ± 9.0	NS	
Body mass index (kg/m^2^) (mean ± S.D.)	22.2 ± 1.9	21.9 ± 2.1	NS	
Duration of infertility (years) (mean ± S.D.)	3.1 ± 2.1	2.9 ± 1.8	NS	
Sperm parameters of partner				
Sperm concentration (×10^6^/mL)	79.9 ± 11.0	77.2 ± 13.0	NS	
Range	21–139	16–153	NS	
Sperm motility (%)	72.9 ± 4.0	77.1 ± 2.4	NS	
Range	67–83	64–81	NS	
Sperm morphology (%)	57.8 ± 5.4	53.9 ± 3.7	NS	
Range	36–75	31–75	NS	

COH-IUI: controlled ovarian hyperstimulation and intrauterine insemination; S.D.: standard deviation; OR: odds ratio, 95% CI: 95% confidence interval; NS: statistically not significant.

**Table 2:** Cycle characteristics of the COH-IUI group (*n* = 281) at the Department of Obstetrics and Gynecology, University of Szeged, from January 2002 to December 2008.

Hormone profile	
Day 3 FSH (mIU/mL)	5.34 ± 2.1
Day 3 LH (mIU/mL)	4.89 ± 3.9
Day 3 E2 (pmol/L)	47.29 ± 40.0
hCG day E2 (pmol/L)	642.8 ± 312.2
hCG day P (ng/mL)	0.39 ± 0.19
hCG day endometrial thickness (mm)	12.11 ± 2.00
Day of hCG administration	9.12 ± 3.01

COH-IUI: controlled ovarian hyperstimulation and intrauterine insemination, expressed as mean ± standard deviation, FSH: follicle-stimulating hormone, LH: luteinizing hormone, E2: estradiol, and P: progesterone.

**Table 3:** Pregnancy characteristics of participants of the COH-IUI group (*n* = 116) and the control group (*n* = 117) at the Department of Obstetrics and Gynecology, University of Szeged, from January 2002 to December 2008.

	Treatment group (COH-IUI) (*n* = 116)		Control group (*n* = 117)		*p* value	OR (95% CI)
CPR (per protocol)	62/116	53.4	45/117	38.5	0.026	1.84 (1.09–3.09)
CPR (endometriosis stages I-II)	31/48	64.6	25/50	50.0	NS	1.82 (0.81–4.10)
CPR (endometriosis stages III-IV)	31/68	45.6	20/67	29.9	NS	1.97 (0.97–4.00)
Miscarriage	2/62	3.2	3/45	6.7	NS	0.47 (0.08–2.92)
Ectopic pregnancy	2/62	3.2	1/45	2.2	NS	1.47 (0.13–16.7)
LBR per protocol	58/116	48.3	41/117	34.2	0.024	1.85 (1.09–3.14)
LBR (endometriosis stages I-II)	30/48	62.5	22/50	44.0	NS	2.12 (0.94–4.76)
LBR (endometriosis stages III-IV)	28/68	41.2	19/67	28.4	NS	1.77 (0.86–3.63)
Multiple pregnancy	4/62	6.5	0/45	0	NS	0.94 (0.88–1.00)

COH-IUI: controlled ovarian hyperstimulation and intrauterine insemination; CPR: cumulative pregnancy rate; LBR: live birth rate; NS: statistically not significant.
